# Indirect Revascularization for Pediatric Moyamoya Angiopathy: Insights from a Brazilian Cohort

**DOI:** 10.3390/jcm14217739

**Published:** 2025-10-31

**Authors:** Alexandre Mello Savoldi, Zeferino Demartini, Mara L. Cordeiro

**Affiliations:** 1 Department of Neurosurgery, Children’s Hospital Pequeno Príncipe, Curitiba 80250-060, PR, Brazil; 2 Faculdades Pequeno Príncipe, Curitiba 80230-020, PR, Brazil; 3 Hospital Universitário Cajuru, Curitiba 80050-350, PR, Brazil; 4 Hospital Universitário Federal do Paraná, Curitiba 80060-900, PR, Brazil; 5 Instituto de Pesquisa Pelé Pequeno Príncipe, Curitiba 80250-060, PR, Brazil; 6 Department of Psychiatry and Biobehavioral Sciences, University of California Los Angeles, Los Angeles, CA 90095, USA

**Keywords:** pediatric stroke, moyamoya disease, moyamoya syndrome, EDAS, EDAMS, indirect revascularization, Latin America

## Abstract

**Background/Objectives**: Moyamoya angiopathy is a progressive occlusive cerebrovascular disorder and a relevant cause of pediatric stroke. While most published data originate from Asian and North American populations, reports from South America remain scarce. This study aimed to characterize the clinical, angiographic, and functional outcomes of pediatric patients with Moyamoya disease (MMD) or Moyamoya syndrome (MMS) who underwent indirect revascularization in a Brazilian cohort, and to contextualize these findings within the international literature. **Methods**: We retrospectively reviewed all patients under 18 years of age who underwent indirect bypass (EDAS/EDAMS) for MMD or MMS at a tertiary pediatric center in Curitiba, Brazil, between 2009 and 2023. Demographic, clinical, and angiographic data were analyzed, including postoperative Matsushima grading, and functional outcomes assessed by the modified Rankin Scale (mRS) and Glasgow Outcome Scale (GOS). All eligible patients received perioperative aspirin (3–5 mg/kg/day). **Results:** Fourteen patients (64% female; mean age 9.9 years) were included: 43% with MMD and 57% with MMS. Hemiparesis (86%), seizures (57%), and cognitive impairment (57%) were the most common symptoms. Most were classified as Suzuki stages II–III. All underwent indirect revascularization; 79% received bilateral procedures. Angiographic follow-up (14 hemispheres) showed good collateral formation (Matsushima grade A: 43%; B: 57%). Complications occurred in three patients (21%), and two (14%)—both MMD—developed new postoperative ischemic strokes despite receiving aspirin therapy. At 3.5 years of mean follow-up, the mean mRS was 1.9 ± 0.8 and the GOS was 4.0 ± 0.3. **Conclusions:** Indirect revascularization in this Brazilian pediatric cohort was feasible and safe, yielding outcomes comparable to international series. Collateral development correlated with functional improvement. These exploratory findings emphasize the importance of early diagnosis, standardized perioperative care, and long-term follow-up, and highlight the need for multicenter collaboration in Latin America.

## 1. Introduction

Moyamoya angiopathy is a rare, progressive, occlusive cerebrovascular disorder involving the terminal portions of the internal carotid arteries and their proximal branches. It is characterized by the gradual narrowing of these vessels and the subsequent development of fragile compensatory collateral networks that appear as a “puff of smoke” on cerebral angiography—a phenomenon first described by Suzuki and Takaku in 1969 [1]. Although considered uncommon worldwide, Moyamoya angiopathy is a significant cause of pediatric cerebrovascular events, accounting for approximately 6% of childhood strokes, with a bimodal age distribution and a slight female predominance [2,3].

Moyamoya disease (MMD) refers to idiopathic and typically bilateral forms, whereas Moyamoya syndrome (MMS) encompasses unilateral or secondary presentations associated with systemic or genetic disorders such as sickle cell anemia, neurofibromatosis type 1, Down syndrome, and cranial irradiation [4]. In pediatric patients, the clinical course is predominantly ischemic, manifesting as transient ischemic attacks, hemiparesis, seizures, or progressive cognitive decline, while in adults, intracranial hemorrhage is more frequently observed [5,6].

Surgical revascularization remains the mainstay of treatment, aiming to prevent recurrent ischemic events and improve cerebral perfusion. Two principal approaches are recognized: direct revascularization, which establishes an immediate extracranial-to-intracranial bypass (e.g., superficial temporal artery to middle cerebral artery anastomosis), and indirect techniques, such as encephaloduroarteriosynangiosis (EDAS) or encephaloduroarteriomyosynangiosis (EDAMS), which promote angiogenesis over time by bringing vascularized tissue in contact with the ischemic brain surface [7,8]. In pediatric populations, indirect procedures are generally preferred because of their technical feasibility in small-caliber vessels and the strong angiogenic potential of the developing brain [9].

Though East Asian, European, and North American centers have reported extensive experience with these conditions, studies from South America are exceedingly scarce. Geographic and genetic variations in Moyamoya disease are gaining increasing recognition, but data from South American populations remain limited [10]. Furthermore, there is still a lack of long-term data on postoperative outcomes and follow-up in low- and middle-income regions [11].

This study presents the first long-term single-center Brazilian series of pediatric patients with MMD and MMS treated with indirect revascularization. By analyzing clinical characteristics, angiographic staging, surgical management, and outcomes, we aim to expand the global understanding of Moyamoya angiopathy and highlight region-specific challenges in the diagnosis and management of this rare but impactful disease.

## 2. Materials and Methods

### 2.1. Study Design and Ethical Approval

This retrospective observational study was conducted at a tertiary pediatric referral hospital in Curitiba, Brazil, from January 2009 to December 2023. The Institutional Ethics Committee approved the study (CAAE: 79457824.0.0000.0097). All procedures were conducted in accordance with the Declaration of Helsinki. Given the retrospective design and use of de-identified data, informed consent was waived. The reporting adhered to the Strengthening the Reporting of Observational Studies in Epidemiology (STROBE) guidelines to ensure methodological transparency and reproducibility.

### 2.2. Patient Selection

We included patients aged <18 years at diagnosis or surgery who had Moyamoya disease (MMD) or Moyamoya syndrome (MMS) and underwent indirect revascularization. Diagnoses were made using the Research Committee on the Spontaneous Occlusion of the Circle of Willis criteria (1997) [2], requiring angiographic evidence of progressive stenosis or occlusion of the distal internal carotid arteries and/or proximal anterior/middle cerebral arteries with abnormal collateral formation. The exclusion criteria were incomplete imaging or clinical data, prior revascularization, or Moyamoya-like changes secondary to acute vasculitis, trauma, or infection. Unilateral and bilateral presentations were eligible; in bilateral cases, each hemisphere was analyzed separately for surgical timing and outcome.

### 2.3. Diagnostic Evaluation

All patients underwent brain MRI/MRA to characterize ischemic burden and vascular anatomy. Digital subtraction angiography (DSA) was performed in every case to confirm diagnosis and to stage severity according to the Suzuki classification. Laboratory work-up included complete blood count, inflammatory markers, autoimmune screening, and hemoglobin electrophoresis to investigate secondary causes (e.g., sickle cell disease). When available, preoperative cerebral perfusion studies (perfusion-weighted MRI, CT perfusion, or SPECT) were obtained to assess hemodynamic compromise and to guide surgical indication. All imaging was jointly reviewed by neurosurgery and neuroradiology to ensure diagnostic consistency. Differential diagnoses (primary vasculitides and other pediatric vasculopathies) were systematically excluded based on clinical, laboratory, and imaging correlation.

### 2.4. Surgical Technique and Perioperative Care

Surgical indications and timing: Indications for surgery included recurrent ischemic symptoms (≥2 clinically documented transient ischemic attacks or strokes), progressive or persistent neurological deficits, and/or radiological evidence of hypoperfusion or diminished cerebrovascular reserve on perfusion studies. After stabilization of acute symptoms and completion of diagnostic work-up, surgery was typically scheduled within 4–8 weeks; in cases with frequent TIAs or marked hemodynamic compromise, earlier intervention was prioritized.

Surgical technique: All operations were performed by the same pediatric neurosurgical team under general anesthesia. Indirect revascularization consisted of encephaloduroarteriosynangiosis (EDAS), encephaloduroarteriomyosynangiosis (EDAMS), or a combination of both. During EDAS, a branch of the superficial temporal artery was dissected and sutured onto the cortical surface through a dural opening. In EDAMS, both arterial and muscular flaps were applied to the ischemic cortex to augment angiogenic stimulus. The technique was selected according to the patient’s vascular anatomy and intraoperative findings. In cases involving bilateral disease, procedures were staged with a minimum interval of 3 months between hemispheres to minimize perioperative ischemic risk. The mean operative time was approximately 3.2 h.

Perioperative management: Postoperatively, patients were monitored in the pediatric intensive care unit for 24–48 h before being transferred to the ward. The average hospital stay was 7 days (range, 5–11). Aspirin was initiated preoperatively in all eligible patients at a dose of 3–5 mg/kg/day and maintained indefinitely unless contraindicated. The rationale for routine aspirin therapy was to reduce perioperative thrombotic risk in accordance with pediatric Moyamoya management guidelines. No patient experienced hemorrhagic complications related to antiplatelet use. Antiepileptic drugs were prescribed only for patients with clinical seizures or electrographic abnormalities.

Postoperative management focused on maintaining normovolemia and normocapnia to prevent ischemic complications. Neurological status was monitored daily during hospitalization, and follow-up neuroimaging was typically performed within 3–6 months after surgery, or earlier if new deficits occurred.

### 2.5. Data Collection and Outcomes

Data extracted from medical records included demographic variables (age, sex), comorbidities (particularly those associated with MMS), presenting symptoms (hemiparesis, seizures, cognitive deficits), angiographic stage, type and laterality of the surgical procedure, postoperative complications (surgical site infection, hematoma, new neurological deficits), and long-term functional outcomes.

Angiographic follow-up was available for all revascularized hemispheres (n = 14) and was performed approximately 3–6 months postoperatively, with repeat studies at 12–18 months in selected cases with persistent or recurrent symptoms. Collateral vessel formation was graded according to the Matsushima system (grades A, B, and C) to evaluate revascularization efficacy.

Functional outcomes were assessed using the modified Rankin Scale (mRS) and the Glasgow Outcome Scale (GOS) at the most recent clinical follow-up. Improvement was defined as a decrease of ≥1 point in mRS or an increase in GOS compared with preoperative status. Functional outcomes were qualitatively correlated with angiographic findings when available, allowing the assessment of the relationship between collateral development and neurological recovery.

### 2.6. Statistical Analysis

Descriptive statistics were used to summarize patient characteristics. Categorical variables were expressed as absolute numbers and percentages, and continuous variables as means with standard deviations or ranges. Comparisons between MMD and MMS groups employed Fisher’s exact test for categorical variables and Student’s *t*-test for continuous variables, after testing for normality with the Shapiro–Wilk test. A *p*-value < 0.05 was considered statistically significant.

Statistical analyses were performed using standard parametric and nonparametric methods. All calculations were independently verified for accuracy. Given the small cohort size, statistical comparisons between MMD and MMS were interpreted cautiously, and the analyses were considered exploratory and hypothesis-generating. Additionally, artificial intelligence (AI) tools were utilized to assist with language refinement and reference formatting during the manuscript preparation. However, all study design, data analysis, interpretation, and final manuscript content were performed and reviewed solely by the authors.

## 3. Results

### 3.1. Patient Demographics and Clinical Presentation

A total of 14 pediatric patients met inclusion criteria and underwent indirect revascularization during the study period. Females predominated (n = 9; 64%), and the mean age at surgery was 9.9 years (range, 2–17 years). Hemiparesis was the most frequently presenting symptom (n = 12; 86%), followed by seizures (n = 8; 57%) and cognitive impairment (n = 8; 57%). Less common manifestations included headache (n = 3; 21%) and visual disturbances (n = 2; 14%).

Demographic and clinical comparisons between Moyamoya disease (MMD) and Moyamoya syndrome (MMS) are summarized in Table 1.

### 3.2. Diagnosis and Angiographic Classification

Six patients (43%) were diagnosed with idiopathic MMD, while eight (57%) were classified as having MMS because they presented with associated comorbidities: sickle cell anemia (n = 3), Down syndrome (n = 2), neurofibromatosis type 1 (n = 1), and two patients with overlapping conditions.

Suzuki staging revealed predominantly moderate disease: stage II (n = 5) and stage III (n = 6), with one patient classified as stage I and two as stage IV.

Postoperative angiographic follow-up was available for all revascularized hemispheres (n = 14) and was typically performed 3–6 months after surgery, with repeat studies conducted at 12–18 months in selected cases. According to the Matsushima grading system, six hemispheres (43%) achieved grade A and eight achieved (57%) grade B collateralization; no instances of grade C were observed.

Patients who achieved Matsushima A/B collateralization generally showed functional improvement (median postoperative mRS 1 vs. 2 preoperatively). Although formal correlation testing was not feasible due to the sample size, this qualitative trend supports the efficacy of indirect revascularization.

Detailed radiological and surgical outcome data are presented in Table 2.

### 3.3. Surgical Procedures and Perioperative Course

All patients underwent indirect revascularization: encephaloduroarteriosynangiosis (EDAS) alone (n = 9), encephaloduroarteriomyosynangiosis (EDAMS) alone (n = 3), or combined procedures (n = 2). Bilateral procedures were performed in 11 patients (79%).

**Figure 1:**
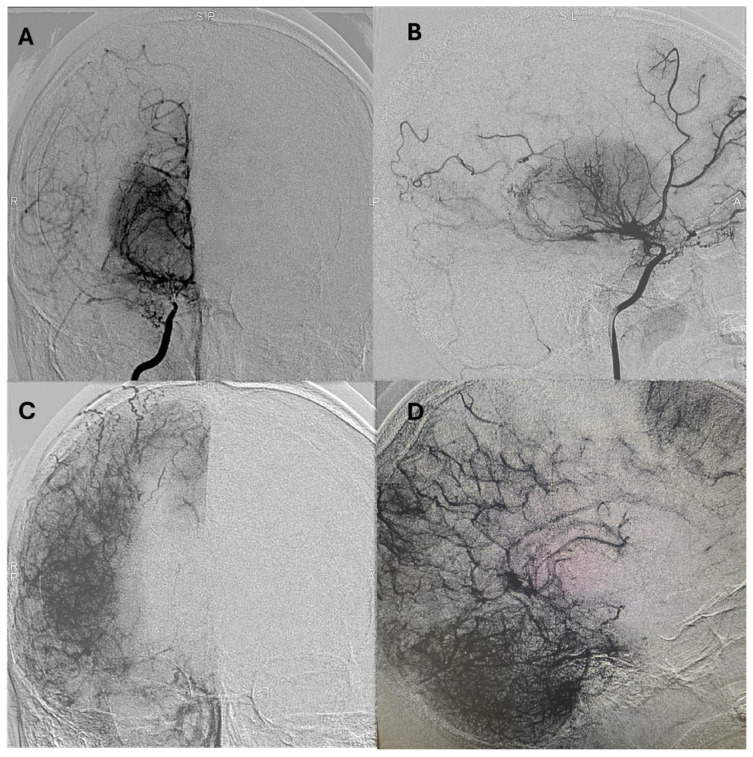
Postoperative digital subtraction angiography (DSA) of the right internal carotid artery (ICA) in anteroposterior (**A**) and lateral (**B**) views demonstrate severe stenosis of the terminal ICA and a thread-like appearance of the M1 and A1 segments. There is reconstitution of the middle cerebral artery (MCA) and anterior cerebral artery (ACA) via lenticulostriate collaterals, consistent with Suzuki stage III. The parenchymal phase (**C**,**D**) reveals a focal hypovascular zone, which is indicative of decreased capillary density and chronic ischemic changes secondary to Moyamoya disease. DSA: digital subtraction angiography; ICA: internal carotid artery; MCA: middle cerebral artery; ACA: anterior cerebral artery.

**Figure 2:**
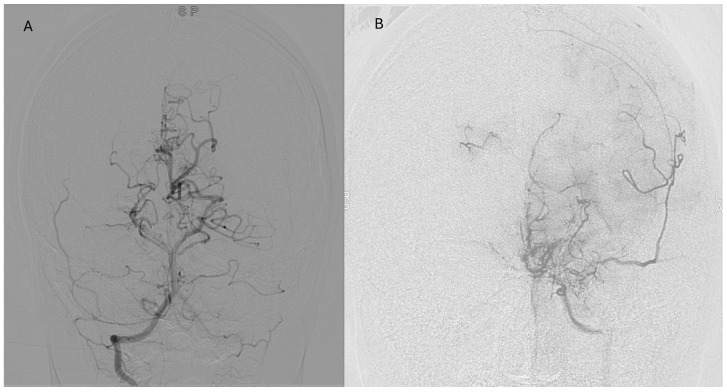
Postoperative anteroposterior DSA of the right vertebral artery (**A**) and left ICA (**B**) following pial synangiosis using the superficial temporal artery as a donor vessel. The images demonstrate robust ingrowth of transdural collaterals. Notably, there is visualization of external carotid artery (ECA) to ICA collaterals arising from the middle meningeal and superficial temporal arteries. These findings are consistent with Moyamoya disease involving posterior circulation. DSA: digital subtraction angiography; ECA: external carotid artery; ICA: internal carotid artery.

**Figure 3:**
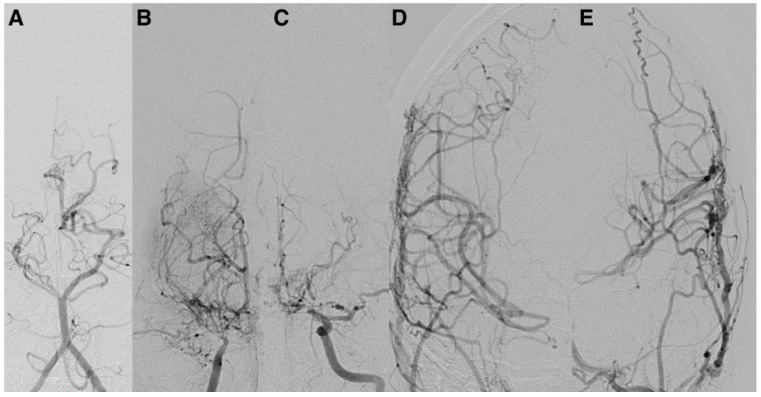
Postoperative anteroposterior DSA in a pediatric patient with Moyamoya disease, demonstrating successful indirect revascularization via encephaloduroarteriosynangiosis (EDAS). There is marked neovascularization originating from the superficial temporal artery, along with reduced opacification of the internal carotid arteries. (**A**) Left vertebral artery; (**B**) right internal carotid artery; (**C**) left internal carotid artery; (**D**) right external carotid artery; (**E**) left external carotid artery. DSA: digital subtraction angiography; EDAS: encephaloduroarteriosynangiosis.

Representative postoperative angiographic images are shown in Figure 1, Figure 2 and Figure 3. The mean operative time was 3.2 h and the average length of hospital stay was 7 days. All patients were monitored in the pediatric intensive care unit for 24–48 h before being transferred to the ward.

Aspirin was administered preoperatively in all eligible patients (3–5 mg/kg/day) and maintained indefinitely unless contraindicated. No hemorrhagic complications related to antiplatelet therapy occurred.

### 3.4. Postoperative Complications

Three patients (21%) developed postoperative complications. Two experienced hemorrhagic events—a subdural hematoma and an intraparenchymal hematoma—both of which were managed conservatively with serial imaging and close monitoring. One patient developed a superficial surgical site infection, which was successfully treated with antibiotics. Additionally, two patients (14%), both with idiopathic MMD, experienced new ischemic strokes during the postoperative period. These were managed with supportive measures, including hydration, blood pressure optimization, and early rehabilitation, without the need for reoperation.

### 3.5. Functional Outcomes and Group Comparisons

At a mean follow-up of 3.5 years, the mean modified Rankin Scale (mRS) score was 1.9 ± 0.8, indicating mild-to-moderate disability with preserved functional independence in most patients. The mean Glasgow Outcome Scale (GOS) score was 4.0 ± 0.3, consistent with overall favorable neurological outcomes.

Comparative outcomes between MMD and MMS subgroups are shown in Table 3.

No statistically significant differences were identified between groups regarding postoperative complications, ischemic events, or functional outcomes (*p* > 0.05). However, the occurrence of new strokes exclusively in the MMD subgroup suggests a possible trend toward more aggressive disease in idiopathic cases, although statistical confirmation was limited by the small sample size.

Given the limited cohort size, all statistical comparisons should be interpreted cautiously, and these findings are considered exploratory and hypothesis-generating.

## 4. Discussion

This study presents the first long-term pediatric Moyamoya case series from Brazil, contributing to the limited data available from Latin America. Although the sample size was small, the demographic and clinical characteristics observed—female predominance, ischemic presentation, and mean age at surgery—were consistent with reports from Asian and North American cohorts [2,3,5]. These parallels suggest that the phenotypic expression of Moyamoya angiopathy in Brazilian children resembles that described in other global populations.

A notable finding was the predominance of Moyamoya syndrome (MMS), which showed a particular association with sickle cell anemia, Down syndrome, and neurofibromatosis type 1. While idiopathic Moyamoya disease (MMD) predominates in East Asian series, Western cohorts typically report a higher proportion of MMS [4,6]. Our data align with these Western trends and emphasize the importance of systematic screening for associated conditions in Brazilian children, as these comorbidities influence prognosis, perioperative risk, and the need for multidisciplinary management.

Indirect revascularization remains the standard surgical approach for pediatric Moyamoya worldwide, favored for its technical feasibility in small-caliber vessels and the high angiogenic potential of children [7,8]. In this series, the complication rate (21%) fell within the 15–30% range described in international pediatric studies [7]. Two new ischemic strokes occurred exclusively in the MMD subgroup [8,12,13]. This finding may reflect the more advanced steno-occlusive stage and poorer baseline collateral network typically observed in idiopathic cases. The delayed development of surgical collaterals after indirect techniques may create a transient “ischemic window,” during which patients remain vulnerable to hypoperfusion-related injury. Furthermore, perioperative hemodynamic instability, hyperventilation, and fluctuations in blood pressure are recognized risk factors for ischemic events. Although our study was underpowered to demonstrate statistical significance, this trend supports the concept of a more aggressive biological course in primary MMD, as previously reported in East Asian and Western cohorts [5,12,14].

Postoperative angiographic follow-up was available for all revascularized hemispheres (n = 14). Examinations were typically performed at 3–6 months after surgery and repeated at 12–18 months in selected cases with persistent or recurrent symptoms. According to the Matsushima grading system, six hemispheres (43%) achieved grade A and 8 (57%) grade B collateralization, with no cases classified as grade C. Patients who developed grade A/B collaterals demonstrated parallel improvement in functional outcomes, with a median postoperative mRS of 1 compared to 2 preoperatively. Although the sample size precluded formal correlation testing, this qualitative relationship reinforces the functional impact of successful indirect revascularization on cerebral perfusion and recovery.

Most patients showed stabilization or improvement in neurological function at long-term follow-up, confirming the durable benefit of indirect bypass procedures. Nevertheless, the variability in follow-up duration and limited access to standardized imaging underscore a structural challenge in low- and middle-income settings: barriers to consistent neuroimaging and neuropsychological monitoring. Establishing structured long-term follow-up programs is essential to more accurately evaluate the evolution of collateral formation and functional recovery.

Aspirin was prescribed in all eligible patients at a dose of 3–5 mg/kg/day and maintained indefinitely unless contraindicated. No patient experienced hemorrhagic complications related to antiplatelet use, and postoperative ischemic events were not temporally associated with therapy discontinuation. These findings support the safety and practicality of long-term aspirin therapy in the perioperative management of pediatric Moyamoya in this setting.

Cognitive impairment was frequent (affecting 57% of patients), highlighting the impact of chronic cerebral hypoperfusion on neurodevelopment [15,16,17]. Our reliance on clinical observation rather than formal neuropsychological testing is a significant limitation. Future Latin American studies should integrate standardized cognitive assessments to better quantify neurodevelopmental outcomes, identify predictors of poor recovery, and guide educational and rehabilitation strategies.

The main limitations of this study include its retrospective design, single-center scope, and small sample size, which limit its statistical power and generalizability. Consequently, all comparisons between MMD and MMS should be interpreted cautiously. The absence of a surgical or medically managed control group further precludes definitive conclusions about the relative efficacy of indirect revascularization compared with alternative strategies. Therefore, the findings should be regarded as exploratory and hypothesis-generating. Despite these constraints, this series offers valuable insight into the feasibility and safety of indirect bypass surgery in a South American context, where heterogeneity in resources and follow-up availability may influence outcomes.

Future multicenter prospective studies are needed to establish standardized surgical indications, timing, and follow-up protocols; to delineate the temporal evolution of postoperative collateral formation; and to identify predictors of neurological and cognitive outcomes. Moreover, genetic and environmental investigations may clarify whether distinctive pathophysiological mechanisms contribute to Moyamoya angiopathy in Latin American populations.

Given the regional disparities in healthcare infrastructure, creating a multicenter Latin American Moyamoya registry is an urgent step toward overcoming small-sample limitations and promoting collaborative research. Such an initiative would facilitate data sharing, harmonize treatment strategies, and support the development of context-specific clinical guidelines.

In summary, this report expands upon the limited knowledge of pediatric Moyamoya angiopathy in South America. Indirect revascularization proved feasible and yielded outcomes comparable to those from high-volume international centers. These findings underscore the importance of early diagnosis, comprehensive etiological evaluation, and multidisciplinary long-term care. Collaborative efforts across Brazil and Latin America are warranted to consolidate regional expertise, optimize care pathways, and improve outcomes for children with this rare cerebrovascular condition.

## 5. Conclusions

In this long-term Brazilian series of pediatric Moyamoya angiopathy, indirect revascularization demonstrated complication rates and functional outcomes comparable to those reported in international cohorts. Although limited by its small sample size and retrospective design, this study confirms the feasibility and safety of indirect bypass procedures in a South American context. The findings highlight the critical importance of early recognition, systematic etiological investigation, and long-term multidisciplinary follow-up. Even in regions with variable access to high-complexity neurosurgical care, indirect techniques remain a practical and effective option for children affected by this rare disease.

By documenting real-world outcomes from an underrepresented region, this study provides a valuable foundation for future research, capacity building, and health policy development in Latin America. Collaborative multicenter efforts and national registries across Brazil and neighboring countries are urgently needed to better characterize disease progression in idiopathic versus syndromic forms, harmonize treatment protocols, and optimize care within resource-variable healthcare systems.

## Figures and Tables

**Table 1:** Sociodemographic and preoperative clinical characteristics of patients with Moyamoya disease (MMD) and Moyamoya syndrome (MMS).

Variable	Moyamoya Disease (MMD)	Moyamoya Syndrome (MMS)	Notes
Number of cases	6 (43%)	8 (57%)	Total: 14 patients
Mean age at surgery (years)	5.0	12.5	Overall mean: 9.9 years
Sex—Female	3 (50%)	6 (75%)	Total: 9 (64%)
Sex—Male	3 (50%)	2 (25%)	Total: 5 (36%)
Laterality	Bilateral: 5, Unilateral: 1	Bilateral: 6, Unilateral: 2	Bilateral in 11 patients (79%)
Preoperative intellectual deficit	3 (50%)	3 (37.5%)	Total: 6 (43%)
Preoperative motor deficit	6 (100%)	6 (75%)	Total: 12 (86%)
Preoperative seizures	5 (83%)	6 (75%)	Total: 11 (79%)
Previous TIA	2 (33%)	5 (62.5%)	Total: 7 (50%)
Previous ischemic stroke	6 (100%)	7 (87.5%)	Total: 13 (93%)
Previous hemorrhagic stroke	0	1 (12.5%)	Total: 1 (7%)

**Table 2:** Radiological, functional, and surgical outcomes of patients with Moyamoya disease (MMD) and Moyamoya syndrome (MMS).

Variable	Moyamoya Disease (MMD)	Moyamoya Syndrome (MMS)	Notes
Imaging findings (CT/MRI)	Right gliosis (4), bilateral (1), left frontal (1)	Gliosis (capsular, frontal, bilateral), 1 normal	Abnormal findings in 13 patients (93%)
Suzuki stage (pre-op)	I–IV (mostly II–III)	I–V (mostly I)	Based on angiographic classification
Matsushima grade (post-surgical)	Grade A: 3. Grade B: 3	Grade A: 3. Grade B: 5	Grade A: 6 (46%). Grade B: 8 (57%)
Surgical technique	Indirect (100%)	Indirect (100%)	EDAS or EDAMS in all cases
Modified Rankin Scale (pre-op)	Mean 1.8 ± 0.4	Mean 2.0 ± 0.8	Functional independence in most patients
Modified Rankin Scale (post-op)	Mean 1.7 ± 0.5	Mean 2.0 ± 0.9	No significant change post-op
Glasgow Outcome Scale (post-op)	Mean 4.0 ± 0.0	Mean 4.1 ± 0.3	Predominantly favorable outcomes
Perioperative complications	3 cases: surgical site infection, subdural hematoma, intraparenchymal hematoma	–	Total: 3 patients (21%)
New postoperative strokes	2 (33%)	0 (0%)	Non-significant trend (*p* = 0.165)

**Table 3:** Comparative analysis of clinical features and outcomes between mmd and mms groups.

Variable	MMD (n = 6)	MMS (n = 8)	*p*-Value
Seizures	5 (83%)	6 (75%)	>0.05
Cognitive impairment	3 (50%)	5 (62%)	>0.05
Surgical complications	1 (17%)	2 (25%)	>0.05
New strokes	2 (33%)	0 (0%)	0.165
mRS at follow-up (mean)	1.7 ± 0.5	1.7 ± 0.5	>0.05
GOS at follow-up (mean ± SD)	4.0 ± 0.0	4.1 ± 0.3	>0.05

Abbreviations: MMD, Moyamoya disease; MMS, Moyamoya syndrome; mRS, modified Rankin scale; GOS, Glasgow Outcome Scale; SD, standard deviation.

## Data Availability

The datasets generated and analyzed during the current study are available from the corresponding author (M.L.C.) upon reasonable request. All data have been anonymized to protect patient confidentiality.
